# Deregulated Nrf2-Keap1-BACH1 axis in autism spectrum disorder

**DOI:** 10.1016/j.redox.2025.103837

**Published:** 2025-08-21

**Authors:** Andrea Vallese, Sara Melija, Joussef Hayek, Alessandra Pecorelli, Giuseppe Valacchi

**Affiliations:** a Dept. of Environmental and Prevention Sciences, University of Ferrara, Ferrara, Italy; b Dept. of Bioscience and Agro-Food and Environmental Technology, University of Teramo, Teramo, Italy; c Toscana Life Science Foundation, Siena, Italy; d Dept. of Food, Bioprocessing and Nutrition Sciences, Plants for Human Health Institute, North Carolina State University, Kannapolis, NC, USA; e Animal Science Dept., Plants for Human Health Institute, North Carolina State University, Kannapolis, NC, USA; f Dept. of Food and Nutrition, Kyung Hee University, Seoul, South Korea

**Keywords:** Neurodevelopmental disorders, Oxidative stress, Heme oxygenase-1, Antioxidant response element (ARE)

## Abstract

Autism Spectrum Disorder (ASD) is a group of neurodevelopmental disorders characterized by impairments in social communication, restricted interests, and repetitive behaviors. Although its etiology remains incompletely understood, increasing evidence suggests a multifactorial origin involving genetic alterations, immune dysregulation, and environmental exposures. The aim of this study was to investigate the redox-sensitive Nrf2 signaling pathway in primary dermal fibroblasts isolated from ASD patients. Our results revealed constitutive activation of Nrf2, accompanied by reduced expression of its downstream target heme oxygenase-1 (HO1) and marked nuclear accumulation of the transcriptional repressor BACH1 in ASD cells. Moreover, ASD fibroblasts failed to increase Nrf2 nuclear translocation upon sulforaphane (SFN) stimulation, a response consistent with elevated basal levels of Keap1, a negative regulator that sequesters Nrf2 in the cytoplasm. Notably, treatment with hemin, known to induce nuclear export and degradation of BACH1, successfully restored HO1 gene and protein expression and ameliorated impaired mitochondrial function in ASD fibroblasts, as suggested by the decrease of mtROS levels and the restored mitochondrial membrane potential. Collectively, these results identify a dysregulation of the Nrf2-Keap1-BACH1 axis in ASD and suggest that pharmacological targeting of this pathway may offer therapeutic potential to correct the redox imbalance associated with the disorder.

## Graphical abstract

**Figure:**
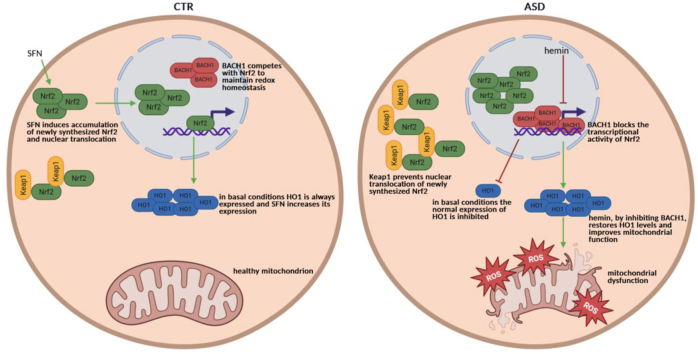

## 1 Introduction

Autism Spectrum Disorder (ASD) is a complex and heterogeneous neurodevelopmental disorder and represents the most significant childhood mental health condition [1,2]. It manifests itself through a variety of symptoms, including impaired social interaction, communication deficits, and restricted, repetitive behaviors and interests [1,3]. These behavioral abnormalities, coupled with sensory sensitivities and rigid adherence to routines, often lead to social isolation, bullying, and psychological distress, including increased vulnerability to self-harm [4]. With an estimated global prevalence rate of 1 in 100 children, ASD poses profound clinical and societal challenges for both individuals diagnosed and their families [5]. The disorder typically develops into a lifelong condition, making it a source of chronic stress for affected families due to the persistence of symptoms, the presence of comorbidities, and limited access to appropriate health care support [6,7].

The etiology of ASD remains a subject of ongoing research, with genetic, environmental, and neurobiological factors all contributing to its pathogenesis [[8], [9], [10]]. Recent studies have highlighted the potential involvement of oxidative stress and neuroinflammation in the development and progression of ASD [[11], [12], [13]]. Elevated levels of oxidative stress and inflammatory biomarkers have been consistently observed in individuals with ASD, and these factors appear to correlate with clinical severity [14]. The interplay between oxidative stress and inflammation, *i.e.*, oxinflammation, particularly their role in disrupting neurodevelopmental processes, is of increasing interest as it may provide insights into the underlying mechanisms of the disorder [[14], [15], [16]]. A dysregulated balance between pro-inflammatory and anti-inflammatory pathways appears to contribute to the pathophysiology of ASD, exacerbating the core symptoms of social and communication dysfunction [17]. Oxidative stress may play a critical role in the etiology of ASD by acting as a mechanism linking genetic, environmental, and immunological factors [18]. Indeed, increasing evidence from the literature suggests that oxidative stress contributes to both the onset and clinical features of ASD [19,20]. In our previous study, we described a deregulation of Nucleotide-binding domain and Leucine-rich-repeat-containing (NLR) family pyrin domain containing 3 (NLRP3) inflammasome activation, mitochondrial dysfunction, and a bidirectional detrimental crosstalk between these components in ASD [21]. In this scenario, the redox-sensitive transcription factor Nuclear factor-erythroid 2 Related Factor 2 (Nrf2) is particularly relevant, as evidence suggests its critical role in oxidative stress, mitochondrial dysfunction and immune/inflammation dysregulation [22]. Nrf2 was identified in 1994 as a regulator of beta-globin gene expression [23]. It has been shown to act as a positive regulator of the human Antioxidant Response Element (ARE), which controls the expression of antioxidant enzymes such as NAD(P)H:quinone Oxidoreductase 1 (NQO1) [24]. Under normal physiological conditions, Nrf2 is sequestered in the cytoplasm through its interaction with Kelch-like ECH-associated protein 1 (Keap1) [25,26]. Keap1, as part of an E3-ligase complex, continuously targets Nrf2 for proteasomal degradation, thereby keeping its activity suppressed [27,28]. However, when Nrf2 activators such as reactive oxygen species (ROS) or compounds like sulforaphane (SFN) are present, they modify cysteine residues on Keap1, leading to a conformational change in the Nrf2-Keap1 complex that prevents Nrf2 ubiquitylation [29,30]. Suppression of Nrf2 ubiquitylation leads to the stabilization and accumulation of newly synthesized Nrf2, allowing its subsequent translocation to the nucleus where it dimerizes with small musculoaponeurotic fibrosarcoma (sMAF) proteins and binds to the ARE in the promoter regions of target genes [31]. This binding promotes the expression of several cytoprotective and antioxidant genes such as *HMOX1*, *NQO1* and *SODs* [29]. At the level of AREs, Nrf2 competes for DNA binding with the transcriptional repressor BTB and CNC homology 1 (BACH1), which regulates the expression of target genes by antagonizing Nrf2 activity [32]. Under basal conditions, sMAF proteins typically form heterodimers with BACH1 [33]. However, ROS-induced elevation of intracellular levels of free heme, which acts as a ligand for BACH1, facilitates dissociation of BACH1 from the AREs. Once bound by heme, BACH1 is exported from the nucleus, allowing Nrf2 to bind to the ARE sequences and initiate transcription [34,35].

Considering the oxinflammatory milieu characterizing ASD, the present study aimed to define the activity of the Nrf2 pathway in primary dermal fibroblasts derived from ASD patients and from healthy controls (CTRs). Specifically, we investigated the activation state of Nrf2 and its functional output by assessing the downstream expression of HO1, a gene under Nrf2 transcriptional regulation [36]. To this end, SFN was used as an Nrf2 activator [37], and hemin was employed to repress BACH1 [35]. Our objective was to identify potential novel targets for understanding the pathophysiology of ASD.

## 2 Materials and methods

### 2.1 Study approval

The study cohort comprised five individuals, aged between 7 and 29 years (3 males and 2 females; average age: 16.25), who had received a clinical diagnosis of ASD. A control group was established, consisting of four neurotypical individuals matched for age and sex (2 males and 2 females; average age: 17.75). All participants with ASD were recruited from the Department of Child Neuropsychiatry at the University Hospital of Siena (Siena, Italy). The research protocol received ethical approval from the Ethics Committee of the Institutional Review Board of the University Hospital, Azienda Ospedaliera Universitaria Senese (AOUS), Siena, Italy [Approval protocol 11615_2010, January 25, 2010]. Written informed consent was obtained from the parents or legal guardians of all participants. The study was conducted in accordance with the ethical standards outlined in the Declaration of Helsinki [21].

### 2.2 Human dermal fibroblasts primary culture

As previously reported [38], primary dermal fibroblasts were isolated from 3-mm skin punch biopsies. Samples were obtained from healthy control subjects (n = 4), either during routine health assessments or through voluntary donation, and from individuals with ASD (n = 5) during scheduled clinical evaluations. Fibroblasts were cultured in low-glucose Dulbecco's Modified Eagle Medium (DMEM; 1.0 g/L glucose; cat. 10-014-CV, Corning, New York, NY, USA) supplemented with 10 % (v/v) fetal bovine serum (cat. 35-011-CV, Corning, New York, NY, USA), and antibiotics (100 IU/mL penicillin and 100 μg/mL streptomycin; cat. 30-002-CI, Corning, New York, NY, USA). Cultures were maintained at 37 °C in a humidified incubator with 5 % CO_2_. All experimental procedures were carried out using fibroblast cultures up to the fifteenth in vitro passage.

### 2.3 Cell treatments

CTR and ASD fibroblasts were seeded at a density of 9000 cells/cm^2^ in complete growth medium [39]. After 24 h of incubation, cells were treated with 10 μM SFN (cat. HY-13755A, MedChemExpress LLC, Princeton, NJ, USA) dissolved in DMSO or vehicle control (DMSO) for 1, 3, 6, 12 or 24 h according to the specific experimental protocol. To promote BACH1 nuclear export and proteasomal degradation, cells were treated overnight with hemin (cat. H9039, Sigma-Aldrich, St. Louis, MO, USA) dissolved in DMSO.

### 2.4 Protein extraction and Western blot (WB) analysis

Total protein extraction was performed according to previously established protocols [40,41]. Briefly, fibroblasts were lysed in Radio-Immunoprecipitation Assay (RIPA) buffer (cat. AAJ62524AD, Alfa Aesar, Tewksbury, MA, USA) supplemented with 1 % (v/v) protease inhibitor cocktail and 1 % (v/v) phosphatase inhibitor cocktail (respectively, cat. 78430 and cat. 1862495, Thermo Fisher Scientific, Waltham, MA, USA). Lysates were incubated on ice for 30 min with intermittent vortexing (5 s every 5 min), followed by centrifugation at 17000 *g* for 30 min at 4 °C. The resulting supernatant containing total cellular proteins was collected for analysis.

For nuclear and cytoplasmic protein fractionation, cell pellets were first resuspended in a cytosolic extraction buffer composed of 10 mM HEPES (pH 7.9), 10 mM KCl, 1.5 mM MgCl_2_, 0.3 % Triton X-100, 0.5 mM dithiothreitol, 0.5 mM phenylmethylsulfonyl fluoride, and protease/phosphatase inhibitors. After 30 min in ice with vortexing (5 s every 5 min), samples were centrifuged at 18000 g for 15 min at 4 °C. The cytoplasmic supernatant was collected, and the nuclear pellet was resuspended in a high-salt nuclear extraction buffer containing 20 mM HEPES (pH 7.9), 0.6 M KCl, 1.5 mM MgCl_2_, 20 % glycerol, 0.5 mM phenylmethylsulfonyl fluoride, and protease/phosphatase inhibitors. Nuclear lysis was facilitated by three freeze-thaw cycles followed by 45 min of shaking at 4 °C. The lysate was then centrifuged at 21000 g for 5 min at 4 °C, and the nuclear protein-containing supernatant was collected.

Protein concentrations were determined using the Quick Start™ Bradford Protein Assay (cat. 5000201, Bio-Rad Laboratories, Inc., Hercules, CA, USA) with bovine serum albumin (BSA) as the standard. Equal amounts of protein were denatured and separated by SDS-PAGE, followed by electrophoretic transfer to nitrocellulose membranes (cat. 1620115, Bio-Rad Laboratories, Inc.). Membranes were blocked with EveryBlot™ Blocking Buffer (cat. 12010020, Bio-Rad Laboratories, Inc.) for 5 min at room temperature and then incubated overnight at 4 °C with primary antibodies diluted in the same buffer (see Table 1). After washing, the membranes were incubated with HRP-conjugated secondary antibodies (see Table 1) for 1.5 h at room temperature, followed by additional washes with Tris-buffered saline (TBS, pH 7.5) containing 0.05 % (v/v) Tween 20 (cat. P5927, Sigma-Aldrich). Immunoreactive bands were visualized using the Clarity™ Western ECL Substrate Kit (cat. 1705060, Bio-Rad Laboratories, Inc.) and imaged with the ChemiDoc™ MP Imaging System (Bio-Rad Laboratories, Inc.) and associated software.

**Table 1:** List of antibodies used in this study.

Antibody	Catalog Number	Dilution	Source
anti-BACH1	sc-271211	WB 1:500	Santa Cruz Biotechnology, Inc. (Santa Cruz, CA, USA)
anti-HO1	sc-390991	WB 1:500	Santa Cruz Biotechnology, Inc. (Santa Cruz, CA, USA)
anti-α-tubulin	sc-23948	WB 1:500	Santa Cruz Biotechnology, Inc. (Santa Cruz, CA, USA)
anti-Nrf2	12721	WB 1:1000 IF 1:250	Cell Signaling Technology, Inc. (Danvers, MA, USA)
anti-Lamin AC	4777	WB 1:1000	Cell Signaling Technology, Inc. (Danvers, MA, USA)
anti-Keap1	CSB-PA012147LA01HU	IF 1:200	CUSABIO TECHNOLOGY LLC (Houston, TX, USA)
Peroxidase-conjugated anti-mouse secondary antibody	170–6515	WB 1:10000	Bio-Rad Laboratories (Hercules, CA, USA)
Peroxidase-conjugated anti-rabbit secondary antibody	170–6516	WB 1:10000	Bio-Rad Laboratories (Hercules, CA, USA)
AlexaFluor™ 488 goat anti-rabbit IgG (H + L)	A11034	IF 1:1000	Thermo Fisher Scientific (Waltham, MA, USA)
AlexaFluor™ 594 goat anti-rabbit IgG (H + L)	A11012	IF 1:1000	Thermo Fisher Scientific (Waltham, MA, USA)

Membranes were stripped and re-probed with antibodies against housekeeping protein as a loading control. Densitometric analysis of band intensities was performed using ImageJ software. Protein levels were normalized to α-tubulin, or Lamin A/C, and results were expressed as arbitrary units (a.u.).

### 2.5 Immunofluorescence (IF) analysis

Fibroblasts were seeded on 10 mm^2^ glass coverslips in 24-well plates at a density of 9000 cells/cm^2^. After 24 h, the cells were subjected to the indicated treatments. At the end of the incubation period, the cells were rapidly rinsed with phosphate-buffered saline (PBS) and fixed with ice-cold absolute methanol for 10 min at 4 °C. Permeabilization was performed with 0.2 % Triton X-100 in PBS for 10 min at room temperature, followed by blocking with 1 % BSA in PBS for 1 h at room temperature. Coverslips were then incubated overnight at 4 °C with primary antibodies (see Table 1) diluted in 0.25 % BSA/PBS. The next day, cells were incubated with fluorochrome-conjugated secondary antibodies (see Table 1, diluted in 0.25 % BSA/PBS) for 2 h at room temperature. Nuclear staining was performed with 4′,6-diamidino-2-phenylindole (DAPI; D1306 Invitrogen ThermoFisher Scientific, Waltham, MA, USA) for 5 min at room temperature. After staining, coverslips were mounted onto glass slides using PermaFluor Aqueous Mounting Medium (cat. TA-006-FM, ThermoFisher Scientific, Waltham, MA, USA), and imaging was performed using a Zeiss Z1 AxioObserver LSM10 confocal microscope equipped with a 40X objective. Digital images were processed and analyzed using ImageJ software. Co-localization analysis was performed using the JACoP plug-in, with results expressed as mean fluorescence intensity for expression analysis and Pearson coefficient for co-localization assessment [42,43].

### 2.6 RNA extraction and real-time RT-PCR analysis

For RNA isolation and subsequent real-time RT-PCR, fibroblasts were seeded at a density of 9000 cells/cm^2^ and treated after 24 h as described in section 2.3. Total RNA was extracted by the phenol-chloroform method, as previously reported [44]. Briefly, after aspiration of the culture medium, cells were lysed directly in PureZOL™ RNA Isolation Reagent (cat. 7326880, Bio-Rad Laboratories, Inc.) and the lysate was transferred to 1.5 mL tubes. Chloroform was added at a ratio of 1:5 (chloroform:PureZOL™), and samples were centrifuged at 12000 g for 15 min at 4 °C to separate the phases. The aqueous phase was collected and mixed with isopropanol (1:2 ratio to PureZOL™) for RNA precipitation, followed by centrifugation at 12000 *g* for 10 min at 4 °C. The resulting RNA pellet was washed with 75 % ethanol (1:1 ratio with PureZOL™), centrifuged at 7500 g for 10 min at 4 °C, air dried under a chemical hood for 3–5 min, and resuspended in nuclease-free water.

RNA concentration and purity were assessed using a BioSpec-nano spectrophotometer (Shimadzu Biotech, Duisburg, Germany). cDNA was synthesized from 1 μg of total RNA using the iScript™ Reverse Transcription Supermix (cat. 1708841, Bio-Rad Laboratories, Inc.). Quantitative PCR was performed using SsoAdvanced™ Universal SYBR® Green Supermix (cat. 1725271, Bio-Rad Laboratories, Inc.) on a CFX Connect Real-Time PCR System (Bio-Rad Laboratories, Inc.) according to the manufacturer's instructions.

Gene expression was quantified using the ΔΔCt method with RPL13A as an endogenous control. PCR cycling conditions were as follows: initial denaturation at 95 °C for 10 min, followed by 39 cycles of 95 °C for 15 s and 60 °C for 60 s. Melt curve analysis was performed to verify amplification specificity (65–95 °C, with 0.5 °C increments every 2 s).

The following primer sequences were used:

HO1 forward: 5′-AAGACTGCGTTCCTGCTCAAC-3’

HO1 reverse: 5′-AAAGCCCTACAGCAACTGTCG-3’

RPL13A forward: 5′-CCTGGAGGAGAAGAGGAAAGAGA-3’

RPL13A reverse: 5′-TTGAGGACCTCTGTGTATTTGTCAA-3’.

### 2.7 Mitochondrial reactive oxygen species production

Mitochondrial reactive oxygen species production was assessed in fibroblasts cultured on glass coverslips using the MitoSOX™ Red (cat. M36008, Thermo Fisher Scientific, Waltham, MA, USA) [21]. Cells were incubated with 5 μM MitoSOX Red at 37 °C for 30 min according to the manufacturer's protocol. After staining, cells were rapidly rinsed with PBS, mounted on glass slides with PermaFluor Aqueous Mounting Medium (cat. TA-006-FM, ThermoFisher Scientific, Waltham, MA, USA), and fluorescence intensity was measured within 2 h at the emission wavelength of 580 nm and excitation wavelength of 510 nm with a confocal microscope. Fluorescence intensity was quantified using ImageJ software to assess relative reactive oxygen species levels.

### 2.8 Mitochondrial membrane potential measurement

Mitochondrial membrane potential was assessed in fibroblasts cultured on glass coverslips using the fluorescent probe MitoTracker™ Orange CMTMRos (cat. M7510, Thermo Fisher Scientific, Waltham, MA, USA) [21]. Cells were incubated with 100 nM dye at 37 °C for 45 min according to the manufacturer's instructions. After staining, cells were rapidly washed with PBS, fixed in 4 % formaldehyde for 10 min at room temperature, and counterstained with DAPI (cat. D1306, Invitrogen ThermoFisher Scientific, Waltham, MA, USA) for 5 min at room temperature to visualize nuclei. Coverslips were then mounted onto glass slides using PermaFluor Aqueous Mounting Medium (cat. TA-006-FM, ThermoFisher Scientific, Waltham, MA, USA) and fluorescence intensity was measured at the emission wavelength of 576 nm and excitation wavelength of 554 nm with a confocal microscope. MitoTracker™ fluorescence intensity was quantified using ImageJ software to assess mitochondrial membrane potential.

### 2.9 Statistics

Statistical analyses were performed using GraphPad Prism version 8 (GraphPad Software, San Diego, CA, USA). Data were analyzed by two-way analysis of variance (ANOVA) followed by Tukey's post hoc multiple comparison test to assess differences between groups. A p-value <0.05 was considered statistically significant. Results are expressed as mean ± standard error of the mean (SEM).

## 3 Results

### 3.1 Increased total Nrf2 protein levels and its nuclear translocation in ASD cells

**Fig. 1:**
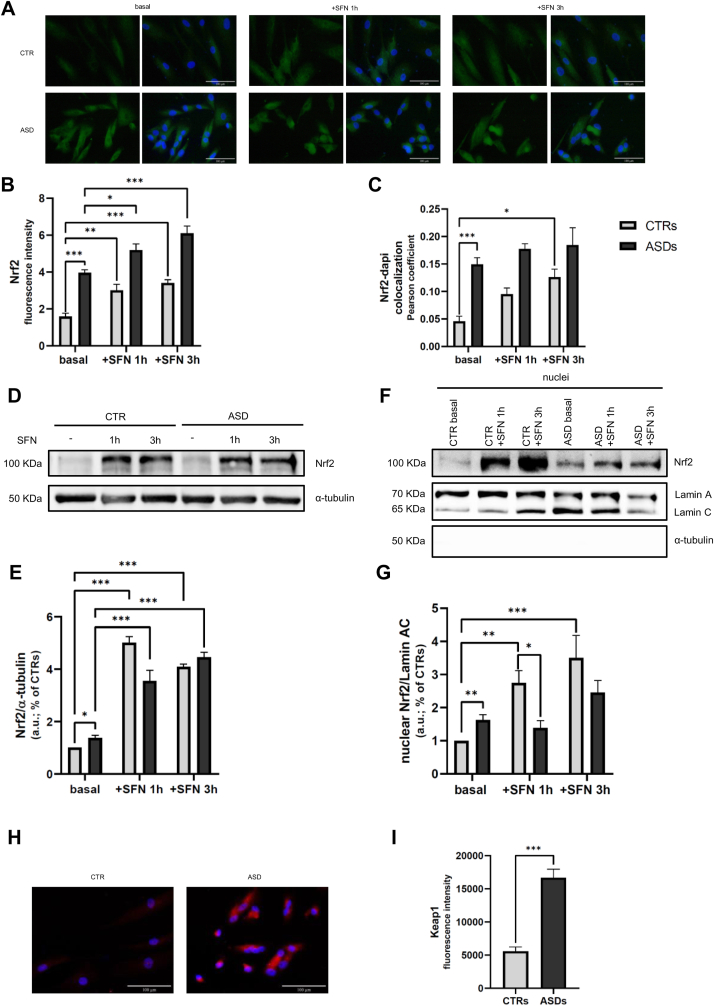
**A** Representative confocal images of three independent experiments of Nrf2 (green fluorescence) in CTR and ASD fibroblasts under basal conditions and stimulated with SFN 10 μM for 1 and 3 h. Nuclei are stained with DAPI (blue fluorescence). Objective 40×. Bar = 100 μm. **B** Analysis of fluorescence intensity of Nrf2 signal. **C** Pearson correlation coefficient values for colocalization of Nrf2 and DAPI. **D-E** Representative image and densitometric analysis of WB of three independent experiments for Nrf2 in CTR and ASD fibroblasts under basal conditions and stimulated with SFN 10 μM for 1 and 3 h. **F-G** Representative image and densitometric analysis of WB of three independent experiments on nuclear fractions for Nrf2 in CTR and ASD fibroblasts under basal conditions and stimulated with SFN 10 μM for 1 and 3 h. **H** Representative confocal images of three independent experiments of Keap1 (red fluorescence) in basal CTR and ASD fibroblasts. Nuclei are stained with DAPI (blue fluorescence). Objective 40×. Bar = 100 μm. **I** Analysis of fluorescence intensity of Keap1 signal. Data were given as means ± SEM. ∗p < 0.05; ∗∗p < 0.005; ∗∗∗p < 0.001.

To evaluate the activation state of the redox-sensitive transcription factor Nrf2, its nuclear translocation was assessed under basal conditions and after exposure to the pro-activating agent SFN for 1 and 3 h. Analyses were performed using both IF and WB on nuclear protein extracts. Fibroblasts derived from ASD patients exhibited significantly increased basal nuclear localization of Nrf2 compared to CTR cells (Fig. 1A, C, F and G). The IF analysis revealed, after 3 h of SFN treatment, a significant increase (higher than 2-fold) in nuclear Nrf2 levels in CTR fibroblasts (Fig. 1 A and C). WB analysis of nuclear fractions showed a significant increase in Nrf2 nuclear localization in CTR cells at both 1 and 3 h (150 % and 250 % of increase, respectively) (Fig. 1F and G). In contrast, no significant change in nuclear translocation was observed in ASD fibroblasts after SFN treatment compared to their basal levels (Fig. 1A, C, F and G). IF and WB analysis on total lysates further confirmed these findings, revealing higher basal Nrf2 expression in ASD cells compared to CTRs (Fig. 1 A, B, D and E). Moreover, SFN stimulation resulted in a significant upregulation of Nrf2 protein expression in both CTR (up to 300 % increase) and ASD fibroblasts (up to more than 150 % increase) (Fig. 1 A, B, D and E).

### 3.2 High levels of Keap1 protein in basal ASD cells

Since SFN treatment did not significantly enhance the nuclear translocation of Nrf2 in ASD fibroblasts, despite an observed increase in total Nrf2 protein levels, we examined the basal expression of Keap1, a key regulator of Nrf2 cytoplasmic retention [25]. As shown in Fig. 1H and I, ASD fibroblasts exhibited significantly increased Keap1 protein expression under basal conditions, with levels approximately three times higher than those observed in CTR cells (p < 0.001).

### 3.3 Reduced HO1 gene and protein expression in ASD fibroblasts and impaired induction by SFN

**Fig. 2:**
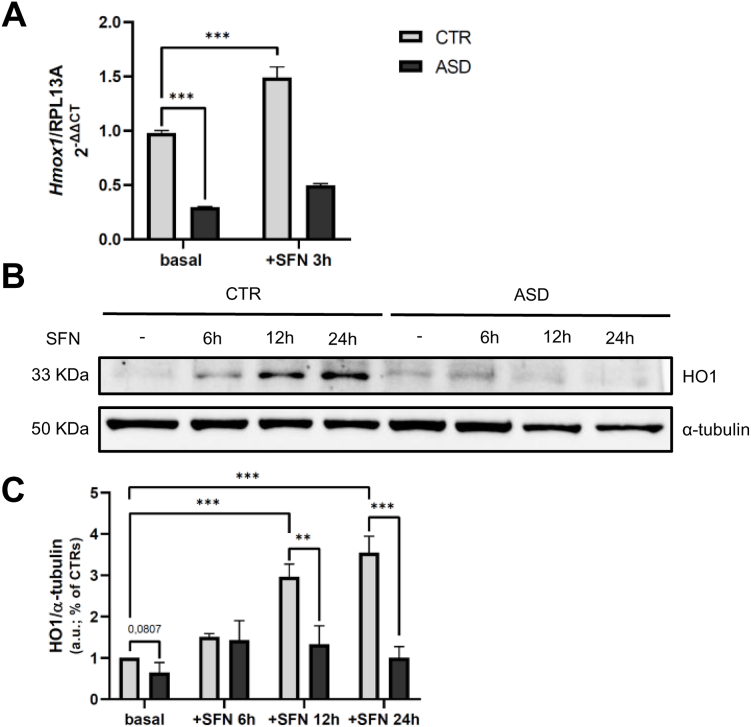
**A***Hmox1* gene expression levels in CTR and ASD fibroblasts under basal conditions and stimulated with SFN 10 μM for 3 h. Data of three independent experiments of real time RT-PCR were given as 2-ΔΔCT, using RPL13A as the reference, and one of the controls as the internal calibrator. **B–C** Representative image and densitometric analysis of WB of three independent experiments for HO1 in CTR and ASD fibroblasts under basal conditions and stimulated with SFN 10 μM for 6, 12 and 24 h. Data were given as means ± SEM. ∗p < 0.05; ∗∗p < 0.005; ∗∗∗p < 0.001.

To investigate the downstream signaling pathway of Nrf2, we analyzed the mRNA expression of *HMOX1* and the protein levels of its encoded product, HO1, a well-characterized transcriptional target of Nrf2 [36]. Quantitative PCR analysis revealed significantly reduced *HMOX1* transcript levels (lower than 75 %) in ASD fibroblasts under basal conditions compared to CTR cells (p < 0.001; Fig. 2A). After 3 h of SFN stimulation, a robust induction of *HMOX1* expression was observed in CTR fibroblasts (+50 %, p < 0.001), whereas ASD cells failed to produce a similar transcriptional response (Fig. 2A). At the protein level, HO1 expression was lower in ASD fibroblasts than in CTRs under basal conditions (50 % lower), although the difference did not reach statistical significance (p = 0.0807). Notably, SFN treatment induced a significant increase in HO1 protein expression in CTR cells at both 12 and 24 h post-treatment (around +200 % and +250 % respectively), whereas ASD fibroblasts showed no significant changes (Fig. 2B and C).

### 3.4 Increased BACH1 nuclear localization in basal ASD cells

**Fig. 3:**
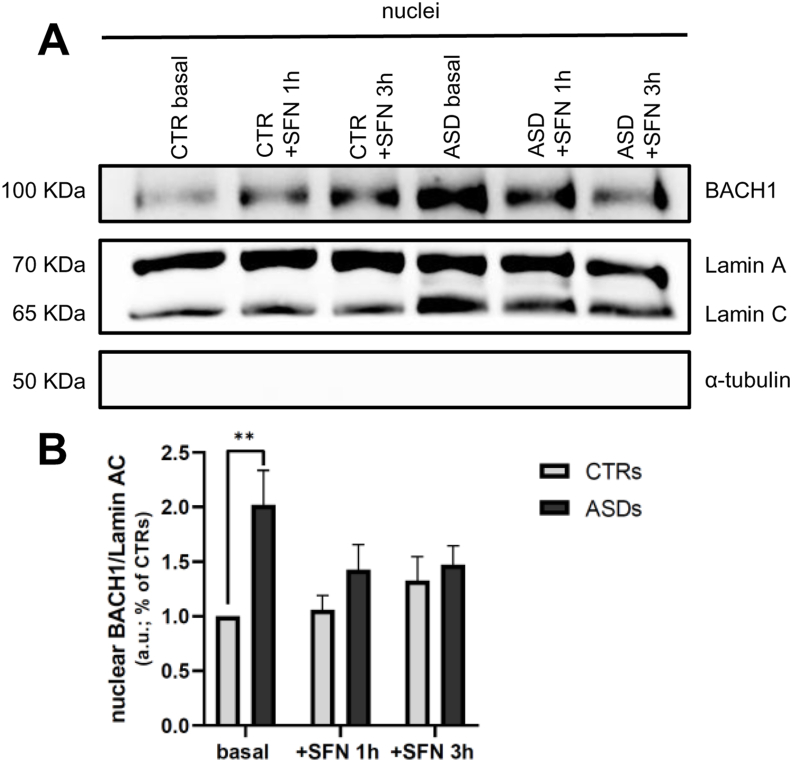
**A-B** Representative image and densitometric analysis of WB of three independent experiments on nuclear fractions for BACH1 in CTR and ASD fibroblasts under basal conditions and stimulated with SFN 10 μM for 1 and 3 h. Data were given as means ± SEM. ∗p < 0.05; ∗∗p < 0.005; ∗∗∗p < 0.001.

Given the observed decrease of HO1 gene and protein expression in ASD fibroblasts, despite elevated nuclear Nrf2 levels, we examined the nuclear localization of BACH1, the primary negative regulator of Nrf2, at the ARE sites [45]. As shown in Fig. 3A and B, ASD fibroblasts exhibited significantly higher nuclear accumulation of BACH1 compared to CTR cells (2-fold higher; p < 0.005). Notably, SFN stimulation did not result in a significant modulation of nuclear BACH1 levels in either CTR or ASD cells.

### 3.5 Hemin treatment promotes BACH1 nuclear exclusion and degradation in ASD cells

**Fig. 4:**
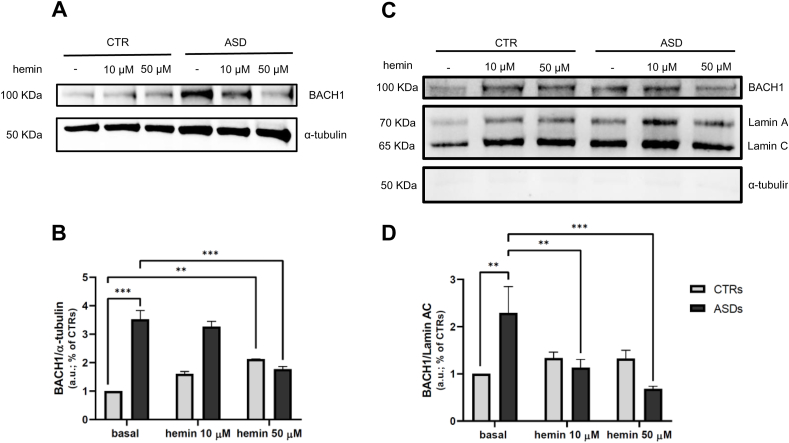
**A-B** Representative image and densitometric analysis of WB of three independent experiments for BACH1 in CTR and ASD fibroblasts under basal conditions and treated overnight with hemin 10 and 50 μM. **C-D** Representative image and densitometric analysis of WB of three independent experiments for BACH1 in nuclear fractions from CTR and ASD fibroblasts under basal conditions and treated overnight with hemin 10 and 50 μM. Data were given as means ± SEM. ∗p < 0.05; ∗∗p < 0.005; ∗∗∗p < 0.001.

Previous studies have shown that hemin facilitates the nuclear export of BACH1 and promotes its ubiquitination and subsequent proteasomal degradation [35,46]. To validate the applicability of hemin in our cellular model, we evaluated its effect on BACH1 protein levels. As shown in Fig. 4A and B, under basal conditions, ASD fibroblasts showed significantly higher total BACH1 protein expression compared to CTRs (p < 0.001). Treatment with 50 μM hemin significantly reduced total BACH1 levels in ASD fibroblasts (p < 0.001), confirming its efficacy in promoting BACH1 degradation. In contrast, the same concentration caused a slight, although significant, increase in total BACH1 levels in CTR cells (p < 0.005) (Fig. 4A and B). WB analysis of nuclear fractions revealed that hemin at both 10 and 50 μM effectively decreased nuclear BACH1 levels in ASD cells, while no significant changes were observed in CTRs (Fig. 4C and D). Consistent with previous findings, nuclear BACH1 levels were significantly elevated in ASD fibroblasts under basal conditions compared to controls (p < 0.005) (Fig. 4C and D).

### 3.6 Hemin treatment restores HO1 gene and protein expression in ASD cells

**Fig. 5:**
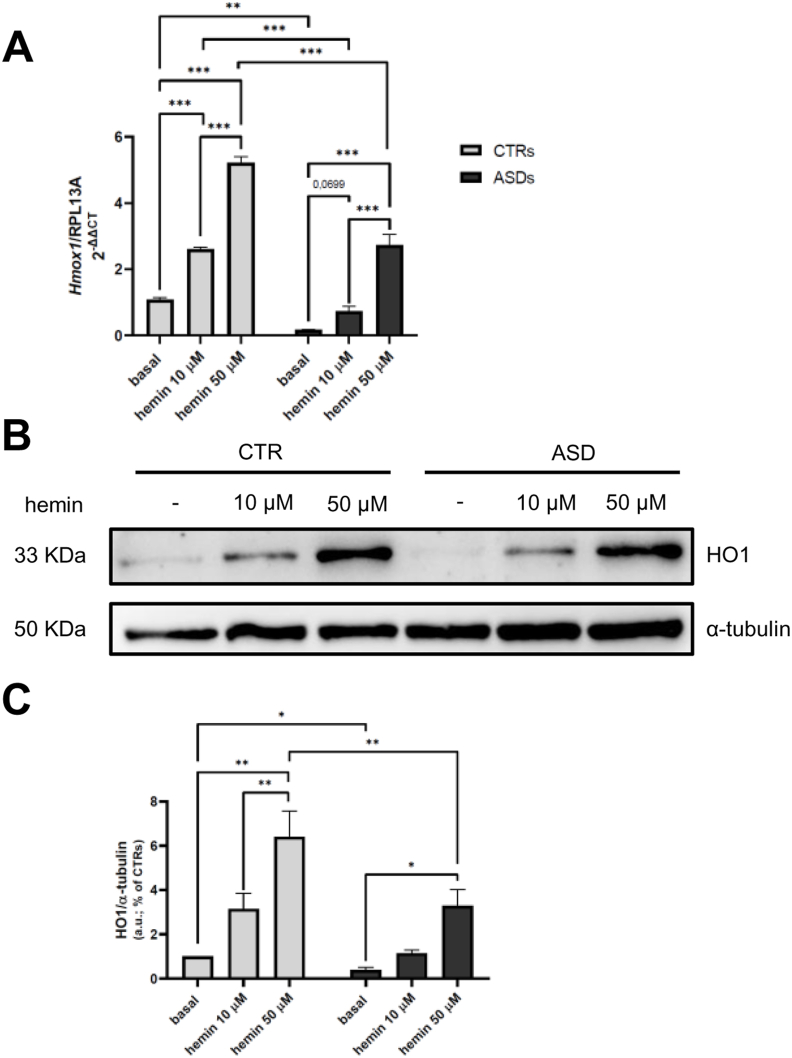
**A***Hmox1* gene expression levels in CTR and ASD fibroblasts under basal conditions and treated overnight with hemin 10 and 50 μM. Data of three independent experiments of real time RT-PCR were given as 2-ΔΔCT, using RPL13A as the reference, and one of the controls as the internal calibrator. **B–C** Representative image and densitometric analysis of WB of four independent experiments for HO1 in CTR and ASD fibroblasts under basal conditions and treated overnight with hemin 10 and 50 μM. Data were given as means ± SEM. ∗p < 0.05; ∗∗p < 0.005; ∗∗∗p < 0.001.

In line with the decrease in nuclear BACH1 after hemin treatment, we observed a concomitant upregulation of HO1 at both the transcriptional and protein levels in ASD cells (Fig. 5 A, B, and C). In ASD fibroblasts, treatment with 50 μM hemin resulted in a significant increase in HO1 gene and protein expression (10 and 6-fold higher, respectively). In addition, although no response in BACH1 nuclear exclusion was observed in CTRs (Fig. 4C and D), both 10 μM and 50 μM hemin significantly increased HO1 gene and protein expression also in these cells (up to more than 200 % and 500 % of increase, respectively). Under basal conditions, ASD cells exhibited significantly lower HO1 gene and protein expression compared to CTRs, further supporting the hypothesis of an impaired antioxidant response in the ASD cellular model.

### 3.7 Restoration of mitochondrial parameters after hemin treatment in ASD cells

**Fig. 6:**
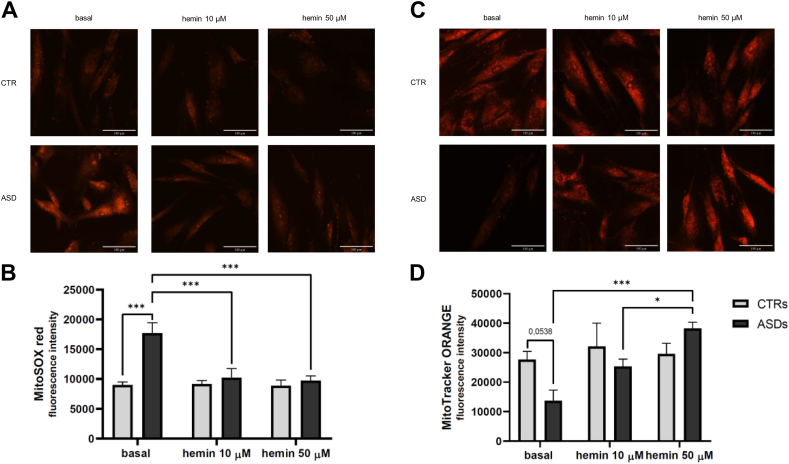
**A-B** Representative confocal images and fluorescence intensity analysis of three independent experiments of MitoSOX Red fluorescence staining of basal CTR and ASD fibroblasts and treated overnight with hemin 10 and 50 μM. **C-D** Representative confocal images and fluorescence intensity analysis of three independent experiments of MitoTracker Orange fluorescence staining of basal CTR and ASD fibroblasts and treated overnight with hemin 10 and 50 μM. Objective 40×. Bar = 100 μm. Data were given as means ± SEM. ∗p < 0.05; ∗∗p < 0.005; ∗∗∗p < 0.001.

Having demonstrated that hemin effectively restores HO1 expression in ASD fibroblasts, we next investigated whether reactivation of the cellular antioxidant defense system could alleviate the redox imbalance typically observed in these cells [21,47]. To do this, mitochondrial reactive oxygen species production was quantified using the MitoSOX fluorescence assay (Fig. 6A and B). Under basal conditions, ASD fibroblasts exhibited significantly elevated mitochondrial reactive oxygen species levels compared to CTRs (p < 0.001). Treatment with hemin, at both concentrations, resulted in a significant decrease of reactive oxygen species production in ASD cells (p < 0.001), indicating a partial restoration of mitochondrial redox homeostasis. Mitochondrial membrane potential was also evaluated after hemin treatment (Fig. 6C and D). Under basal conditions, ASD fibroblasts exhibited a decreased mitochondrial membrane potential compared to CTR cells (lower than 60 %), indicating impaired mitochondrial function. Hemin treatment, especially at the 50 μM concentration, significantly restored the membrane potential levels in ASD cells (p < 0.001), suggesting a partial recovery of mitochondrial integrity.

## 4 Discussion

Although the etiology of ASD remains poorly understood, a growing body of evidence implicates immune-inflammatory dysregulation as a key contributor to its pathophysiology [48]. Furthermore, several studies, including our group, have clearly reported evidences of a redox imbalance in ASD, often in association with mitochondrial dysfunction [21,47,49]. Given the pivotal role of mitochondria in both redox homeostasis and immune signaling, their impairment is thought to promote a pathological state defined OxInflammation [50], characterized by a detrimental interplay between oxidative stress and chronic systemic inflammation [51]. In this context, the transcription factor Nrf2, a key regulator of cellular redox homeostasis [52], emerges as a pathway of particular interest since its dysregulation could contribute to the redox imbalance observed in ASD. This hypothesis is further supported by recent findings suggesting a central role of Nrf2 dysregulation in ASD, with implications for redox imbalance, immune and mitochondrial dysfunction [53]. To explore this aspect, we used primary dermal fibroblasts derived from both ASD patients and healthy controls, a well-established model for studying human disease, as previously demonstrated in Alzheimer's disease, Parkinson's disease, and Rett syndrome [[54], [55], [56]]. Fibroblasts were chosen because of their ease of isolation through minimally invasive procedures and their genetic profile, which closely resembles that of other cell types, including neurons, thus providing a genetically accurate representation of the organism as a whole [57]. The aim of this study was to investigate the potential involvement of the redox-sensitive Nrf2 pathway in the pathophysiological mechanisms underlying ASD.

Through IF and WB analyses performed on total lysates and nuclear fractions, we observed a significantly higher total and nuclear level of Nrf2 in ASD fibroblasts compared to CTRs, in agreement with previous findings [58]. Similar alterations in systemic Nrf2 levels have also been observed in peripheral blood from ASD patients, indicating that such dysregulation is not limited to in vitro models but may reflect broader systemic redox impairments [59]. According to the currently accepted model, redox imbalance induces modifications of Keap1 cysteine residues, resulting in inhibition of Nrf2 degradation, *de novo* Nrf2 accumulation, and its nuclear translocation [60]. Given the well-documented presence of oxidative stress in ASD [18,61], one plausible interpretation of our results is that redox imbalance alters the Keap1-Nrf2 complex conformation, leading to the intracellular and nuclear accumulation of Nrf2. However, our data also indicate that, unlike CTR cells, ASD fibroblasts fail to significantly enhance Nrf2 nuclear translocation upon stimulation with SFN. This observation is consistent with previous reports showing that ASD fibroblasts are unable to increase Nrf2 nuclear translocation in response to oxidative stimuli, such as hydrogen peroxide [47]. We examined the expression of Keap1, the cytoplasmic anchor of Nrf2 [25], after observing that SFN treatment raises total Nrf2 levels but does not promote its nuclear translocation in ASD cells. IF analyses revealed a significant upregulation of Keap1 expression in ASD fibroblasts compared to CTRs, in line with previous findings reporting increased Keap1 levels in the serum of ASD children [58]. Based on these observations, we propose that ASD fibroblasts exhibit a constitutively elevated Keap1 expression, which sequesters Nrf2 in the cytoplasm, preventing its nuclear translocation. This mechanism would explain why SFN treatment results in an increase in total Nrf2 levels without promoting its nuclear accumulation. Indeed, SFN induces a conformational change in Keap1 that prevents Nrf2 ubiquitination and degradation [62], leading to cytoplasmic accumulation of newly synthesized Nrf2. Nevertheless, due to excessive Keap1 levels, this new Nrf2 remains sequestered in the cytoplasm rather than translocating to the nucleus. This explanation would seem in contrast with what is observed under basal conditions, since ASD cells paradoxically show higher levels of nuclear Nrf2 than CTR fibroblasts. This apparent contradiction may be explained by the establishment of a dynamic homeostatic equilibrium: despite the excessive cytoplasmic retention exerted by Keap1, persistent oxidative stress in ASD cells probably drives continuous Nrf2 production, allowing a fraction of it to escape sequestration and translocate into the nucleus. This balance may reflect an adaptive, albeit dysregulated, cellular attempt to counteract the chronic redox imbalance characteristic of ASD. It is important to note that this model remains hypothetical and requires further experimental validation, although supported by the current study.

Due to the constitutive nuclear localization of Nrf2 observed in ASD fibroblasts under basal conditions, we investigated its functional activation by examining the gene and protein expression levels of HO1, a well-characterized downstream target of Nrf2 [36]. Consistent with the impaired ability of ASD cells to promote Nrf2 nuclear translocation upon SFN stimulation, these cells, in contrast to CTRs, did not exhibit a significant increase in HO1 expression following treatment. Unexpectedly, we also found that under basal conditions, both HO1 mRNA and protein levels were significantly lower in ASD fibroblasts compared to CTRs. The low basal expression of HO1, as well as the failure to upregulate its expression in response to oxidative stimuli, has been previously reported in ASD [47,58]. These results indicate that despite elevated levels of nuclear Nrf2, in ASD cells under unstimulated conditions, there is not a corresponding upregulation of HO1, suggesting a functional uncoupling between the nuclear presence of Nrf2 and its transcriptional activity.

To further investigate the observed incongruence between Nrf2 nuclear accumulation and lack of downstream target gene transcription in ASD fibroblasts, we examined the nuclear levels of BACH1, a known transcriptional repressor and functional antagonist of Nrf2 [32]. Under basal conditions, nuclear levels of BACH1 were significantly higher in ASD cells compared to CTRs. These findings support the hypothesis that in ASD fibroblasts, BACH1 may inhibit Nrf2-driven transcription, thereby preventing the expression of downstream target genes such as HO1. To experimentally test this hypothesis, we used hemin, a compound known to promote nuclear export and subsequent degradation of BACH1 [35,46,63].

We first aimed to validate in our cellular model the ability of hemin to induce BACH1 degradation and, in particular, its nuclear export. Consistent with previous findings, our data showed that hemin treatment led to a reduction of both total and nuclear BACH1 levels in ASD fibroblasts, with the most significant effect observed at the 50 μM concentration. In contrast, CTR fibroblasts showed a significant increase in total BACH1 expression with hemin 50 μM and no significant changes in BACH1 nuclear localization after hemin treatments. This response may reflect a compensatory regulatory mechanism, as BACH1 plays a physiological role in modulating Nrf2 activity to maintain transcriptional homeostasis [45]. Accordingly, CTR cells may restore BACH1 expression via feedback mechanisms after hemin-induced degradation to maintain this balance. Therefore, after an initial phase of nuclear extrusion and degradation of BACH1 resulting in HO1 accumulation (visible after the overnight hemin treatment), CTR cells would undergo a physiological process of restoring total and nuclear levels of BACH1 to maintain cellular homeostasis. Importantly, hemin treatment in ASD fibroblasts resulted in a dose-dependent restoration of HO1 gene and protein expression, supporting the role of BACH1 as a transcriptional repressor of the Nrf2 pathway in these cells. These results suggest that the pathological accumulation of nuclear BACH1 in ASD cells contributes to the impaired expression of Nrf2 downstream targets and that its pharmacological removal can partially rescue this dysfunction.

This study uncovers novel aspects of ASD pathophysiology by highlighting a dysregulation of the Nrf2 pathway that contributes to impaired cellular antioxidant defenses and mitochondrial dysfunction in ASD fibroblasts. While our findings provide important mechanistic insights, it is important to acknowledge the limitations of our work. Firstly, the exclusive use of an in vitro model. Although consisting in primary cells isolated from patients, do not allow for a direct correlation with ASD-specific clinical phenotypes. In addition, considering the complexity of ROS chemistry and the technical limitations of current detection methods [64,65], a possible limitation concerns the use of the MitoSOX probe, which might be also affected by several cellular parameters, including plasma and mitochondrial membrane potentials, and mitochondrial morphology and mass [66]. Furthermore, MitoSOX oxidation yields both ethidium, a non-specific oxidation product, and 2-hydroxyethidium, the specific product of the superoxide interaction. So when extrapolating the results we need to take into consideration the overlapping fluorescence spectra of these two species [67]. Despite these challenges, MitoSOX remains a widely used and valuable tool in redox biology due to its relative specificity and practicality in detecting mitochondrial superoxide [68,69].

Finally, we believe that our data could suggest BACH1 and Keap1 as possible new targets to improve ASD clinical features, but clinical and preclinical studies are needed to confirm our hypothesis. Supporting this translational relevance, a recent preclinical study demonstrated that pharmacological activation of Nrf2 using dapagliflozin ameliorated ASD-like behavioral and biochemical alterations in a rodent model, further underscoring the therapeutic potential of targeting this pathway in ASD [70].

## CRediT authorship contribution statement

**Andrea Vallese:** Writing – original draft, Investigation, Formal analysis, Data curation, Conceptualization. **Sara Melija:** Methodology, Formal analysis, Data curation. **Joussef Hayek:** Writing – review & editing, Formal analysis, Conceptualization. **Alessandra Pecorelli:** Writing – original draft, Resources, Project administration, Formal analysis, Data curation, Conceptualization. **Giuseppe Valacchi:** Writing – review & editing, Validation, Supervision, Resources, Funding acquisition, Conceptualization.

## Declaration of competing interest

The authors declare that they have no known competing financial interests or personal relationships that could have appeared to influence the work reported in this paper.

## Data Availability

Data will be made available on request.
